# Supplementary material for: Prevalence of primary dysmenorrhea and its associated factors among adolescent girls in a rural setting of Pakistan
Source: PLOS Glob Public Health. 2026 Mar 26;6(3):e0006162. doi: 10.1371/journal.pgph.0006162 (PMC13020788; doi:10.1371/journal.pgph.0006162)
Supplement: Attachment — Submitted filename: comment.docx [file pgph.0006162.s002.docx]

This is a well-conducted cross-sectional study addressing an important and under-researched topic in a rural Pakistani setting. The manuscript is clearly written, methods are generally robust, and the findings contribute meaningfully to the literature on menstrual health in low-resource contexts. I recommend acceptance after minor revisions addressing the points below.

Comments

Methods

Measurement of dysmenorrhea:The outcome is assessed with a single yes/no question (“Do you have pain during menstruation?”). This may not capture severity, frequency, or functional impact. Please justify this approach or acknowledge it as a limitation. If possible, consider referencing whether pain intensity or days affected were recorded, even if not used in analysis.

Sampling: It is stated that 405 adolescent girls were eligible from the original sample. Please clarify how many were approached vs. participated, and mention any exclusion due to missing data.

Sleep and physical activity measures: Briefly describe how these were defined/collected (e.g., self-report, recall period).

Results

Table 3: Some CIs cross 1 but are marked with asterisks (e.g., eldest child, carbohydrate intake). Please ensure footnote clearly explains that asterisks indicate p<0.1 or p<0.2, not statistical significance at 0.05.

Family history of PD shows a very high OR (~22). Please check for possible over-adjustment or collinearity in the model.

Discussion

The comparison with prevalence in Iran (85%), India (75%), etc., is useful, but note differences in measurement tools and age ranges where possible.

The public health implications are well laid out, but consider adding a sentence on how interventions could be delivered given the high out-of-school rate (e.g., community health workers, mobile clinics).

Language & Clarity

Minor grammatical edits needed (e.g., “post-menarchael” → “post-menarcheal”; “infrequent pad changing (>6 hours)” could be phrased more clearly as “changing pads less frequently than every 6 hours”).

Ensure consistency in reporting confidence intervals: sometimes written as [54–66], elsewhere as [54% to 66%].
