# Supplementary material for: Prevalence of primary dysmenorrhea and its associated factors among adolescent girls in a rural setting of Pakistan
Source: PLOS Glob Public Health. 2026 Mar 26;6(3):e0006162. doi: 10.1371/journal.pgph.0006162 (PMC13020788; doi:10.1371/journal.pgph.0006162)
Supplement: Attachment — Submitted filename: response sheet.docx [file pgph.0006162.s004.docx]

Dear Editor,

I am pleased to submit our manuscript entitled *“Prevalence of Primary Dysmenorrhea and Its Associated Factors among Adolescent Girls in Rural Sindh, Pakistan”* for consideration in **PLOS Global Public Health**.

This study investigates the burden of primary dysmenorrhea among adolescent girls in rural Sindh and explores sociodemographic and lifestyle factors associated with this condition. Despite its high prevalence and impact on adolescent health and education, dysmenorrhea remains under-researched in low-resource rural contexts. Our findings provide evidence that can inform culturally sensitive interventions, strengthen adolescent reproductive health programs, and guide policy development in Pakistan and similar settings.

We believe this manuscript is well-suited for PLOS Global Public Health because:

- It addresses a neglected aspect of adolescent health in rural South Asia, contributing to global equity in research.
- It highlights actionable factors that can be targeted through community-based health education and policy initiatives.
- It aligns with the journal’s mission to amplify diverse voices and evidence from underrepresented regions.

The manuscript is original, has not been published elsewhere, and is not under consideration by another journal. All authors have approved the final version and consent to its submission.

We respectfully request your consideration of this manuscript for publication. Thank you for your time and attention.

Sincerely,
Dr Jai K Das

Associate Director

Institute for Global Health and Development

Associate Professor

Department of Paediatrics and Child Health

Aga Khan University, Karachi, Pakistan

Email: [jai.das@aku.edu](mailto:jai.das@aku.edu)

Response Sheet

| Reviewer 1’s comments |  |
| --- | --- |
| Comment | Response |
| The abstract and results section could be more concise and focused on the most salient findings. | We thank the reviewer for this suggestion. The abstract has been carefully reviewed and structured to highlight the most important findings of the study, including prevalence and key associated factors such as BMI, lifestyle, diet, and menstrual hygiene. We ensured that the abstract remains concise, focused, and within the journal’s recommended word limit while providing a clear summary of the study’s results and public health implications. |
| In the Introduction, You have mentioned ”Nutritional deficiencies, including omega-3 fatty acids, magnesium, vitamin B6, calcium, and iron may further exacerbate PD”. Then you have cited one reference. I suggest that to use the references for each nutritional items that you have listed. For example for omega-3 fatty acid cite this article: Effect of Clupeonella grimmi (anchovy/kilka) fish oil on dysmenorrhea https://doi.org/10.26719/2010.16.4.408. | We thank the reviewer for this helpful suggestion. We agree that providing more specific references strengthens the scientific basis of the statement. The Introduction has been revised to include additional citations supporting the role of individual nutrients—such as omega-3 fatty acids, magnesium, vitamin B6, calcium, and iron—in the context of primary dysmenorrhea. The suggested reference on omega-3 fatty acids has been incorporated, along with relevant literature for the other listed nutrients, to improve clarity and accuracy. |
| The outcome variable (PD) was assessed using a single yes/no question: “Do you have pain during menstruation?” This is a major limitation, as it does not differentiate between primary and secondary dysmenorrhea, assess pain severity, frequency, or functional impact. The authors should clearly acknowledge this as a limitation and consider validating the measure against established tools (e.g., Visual Analog Scale, Menstrual Symptom Questionnaire). | We thank the reviewer for this important comment. This concern overlaps with the other reviewer comment regarding the measurement of primary dysmenorrhea, which we have addressed in detail. We have now clearly and explicitly acknowledged in the Limitations section that the use of a single binary self-reported question does not capture pain severity, frequency, duration, or functional impact.  To improve the validity of the outcome measure, we distinguished primary from secondary dysmenorrhea by excluding respondents with key clinical features suggestive of secondary causes, as specified in the Methods. However, we acknowledge that the measure was not validated against established pain assessment tools such as the Visual Analog Scale or Menstrual Symptom Questionnaire, and such validation was not feasible retrospectively given the secondary nature of the data. This limitation has now been clearly stated in the manuscript. |
| BMI and PD: The finding that overweight/obese girls had lower odds of PD contradicts much of the literature. While the authors propose possible biological and dietary mechanisms, these are speculative and not directly tested. | We thank the reviewer for this important observation. We agree that the inverse association between higher BMI and primary dysmenorrhea observed in our study contrasts with findings from some previous studies. As this was an observational, cross-sectional analysis, we did not directly assess biological or dietary mechanisms underlying this association. We have therefore revised the Discussion to clearly frame the proposed explanations as hypothetical and exploratory rather than causal.  We now emphasize that this finding should be interpreted cautiously and may reflect contextual factors specific to the study population, including nutritional patterns, body composition differences, or residual confounding. We also highlight the need for longitudinal and mechanistic studies to further investigate the relationship between BMI and primary dysmenorrhea in similar settings. |
| Categorization and Measurement of Key Variables: Menarche Age: Categorized only as ≤12 vs. >12 years, which may obscure nuanced age-related risk patterns. | We thank the reviewer for this comment. Age at menarche was derived from secondary data, and we were therefore constrained by the original survey design. In the primary dataset, age at menarche was collected in four predefined categories. For analytical feasibility and to ensure adequate cell sizes, these categories were subsequently combined into ≤12 and >12 years. We acknowledge that this dichotomization may obscure more nuanced age-related risk patterns and have explicitly noted this as a limitation in the manuscript. Future studies using primary data collection could benefit from more granular measurement of age at menarche to better explore dose–response relationships. |
| Sleep and Physical Activity: Definitions (e.g., “inadequate sleep”) are not clearly justified or referenced. Please provide clearer operational definitions and, where feasible, consider more granular categorizations. | We thank the reviewer for this important comment. Sleep duration and physical activity were assessed using standardized items derived from the WHO Health Behaviour in School-aged Children (HBSC) and Global School-based Student Health Survey (GSHS) instruments. In the present study, adequate sleep was operationally defined as self-reported sleep duration of 6–8 hours per night, consistent with the response categories available in the survey and with prior adolescent health studies conducted in similar low- and middle-income settings. Physical activity was classified as adequate if adolescents reported engaging in at least 180 minutes of moderate-to-vigorous physical activity per week, reflecting the minimum threshold captured by the survey instrument and aligned with GSHS-based classifications. We have added these methodological details in the draft. |
| The discussion of middle-born children and emotional stress is speculative and lacks support from the study's qualitative or quantitative data. Either remove these speculative interpretations or frame them as hypotheses for future research. | We thank the reviewer for this important comment. We agree that the discussion of emotional stress among middle-born children is speculative and was not directly assessed in our study. We have therefore revised the Discussion to explicitly frame these interpretations as potential hypotheses rather than causal explanations. The revised text now clearly states that these interpretations are exploratory and highlights the need for future qualitative and longitudinal research to examine psychosocial pathways underlying birth-order differences in primary dysmenorrhea. |
| Some confidence intervals cross 1.0 (e.g., middle-born AOR = 1.7 [0.8–3.4]), yet are described as “significant” or “more likely.” Use more cautious language and distinguish between statistical and clinical significance. | We thank the reviewer for this important point. In this study, statistical significance was defined at a p-value threshold of <0.10, as specified in the Methods and table footnotes, while confidence intervals are presented at the 95% level. As a result, some estimates with p < 0.10 may have 95% confidence intervals that include unity, which can appear inconsistent if interpreted using a conventional 0.05 threshold.  To address this, we have revised the manuscript text to use more cautious language (e.g., “associated with higher odds” rather than “significant” where appropriate) and clarified the distinction between statistical significance and the magnitude or potential clinical relevance of the associations. The table footnotes already specify the significance thresholds used, and we have ensured consistency between the text and tables to minimize ambiguity. |
| Some tables (e.g., Table 1) are overly detailed and could be streamlined or moved to supplementary materials. Do not repeat the results in the text and tables. | Table 1 has been concised to include only the most relevant descriptive information, reducing unnecessary detail while retaining clarity. We have ensured that key findings are not repeated in both the text and tables, improving overall readability. |
| The discussion could be better organized to prioritize the most robust and actionable findings. | We have now worked on the discussion section. |
| Reviewer 2 Comments |  |
| Measurement of dysmenorrhea:The outcome is assessed with a single yes/no question (“Do you have pain during menstruation?”). This may not capture severity, frequency, or functional impact. Please justify this approach or acknowledge it as a limitation. If possible, consider referencing whether pain intensity or days affected were recorded, even if not used in analysis. | We thank the reviewer for this important observation. Primary dysmenorrhea was assessed using a binary (yes/no) self-reported question on menstrual pain, consistent with the structure of the survey instrument and the need for feasibility in large-scale, field-based data collection. To improve the validity of the outcome measure, we distinguished primary from secondary dysmenorrhea by excluding respondents with key clinical features suggestive of secondary causes, as specified in the Methods. However, we acknowledge that this approach does not capture important dimensions of dysmenorrhea, such as pain severity, duration, frequency, or functional impairment. Information on pain intensity or the number of days affected was not available for analysis. We have now explicitly acknowledged this limitation in the manuscript and revised the Discussion to clarify that, despite efforts to enhance outcome validity, the use of a single-item measure may oversimplify symptom experiences. |
| It is stated that 405 adolescent girls were eligible from the original sample. Please clarify how many were approached vs. participated, and mention any exclusion due to missing data. | We thank the reviewer for this clarification request. All 405 adolescent girls who met the eligibility criteria were approached and included in the study sample; there was no non-response at the eligibility stage. Of these, 10 participants (2.5%) had missing values in nutrient intake variables. This proportion was very low and no participants were excluded from the analysis due to missing data. As described in the statistical analysis section, missing data were assessed for a Missing Completely at Random (MCAR) pattern and handled using multiple imputation. Several imputed datasets were generated, and pooled estimates were used in the final analyses to account for uncertainty related to missingness. We have clarified this information in the manuscript to improve transparency. |
| Sleep and physical activity measures: Briefly describe how these were defined/collected (e.g., self-report, recall period). | We thank the reviewer for this suggestion. We have revised the Methods section to explicitly clarify that lifestyle behaviours, physical activity, sleep patterns, and tobacco use were collected using standardized self-reported questionnaire items from the HBSC and GSHS tools with a seven-day recall period. We have also clarified that depression was assessed using the validated Beck’s Depression Inventory. This information has now been added to improve methodological transparency. |
| Table 3: Some CIs cross 1 but are marked with asterisks (e.g., eldest child, carbohydrate intake). Please ensure footnote clearly explains that asterisks indicate p<0.1 or p<0.2, not statistical significance at 0.05. | We thank the reviewer for this comment and appreciate the opportunity to clarify. In Table 3, the footnote already specifies the significance thresholds applied in this study. Variables marked with asterisks correspond to those meeting the predefined levels of statistical significance used in our analysis, where p < 0.10 was considered statistically significant. Variables with p < 0.25 were retained for model building purposes, consistent with standard epidemiological practices for multivariable regression.  Although some confidence intervals cross unity, these estimates correspond to p-values < 0.10, reflecting our chosen significance threshold rather than the conventional 0.05 level. This approach was applied consistently across analyses and is explicitly stated in the table footnote and Methods section. To avoid any potential ambiguity, we have further refined the table footnote to clearly reiterate the interpretation of asterisks and the significance levels used in this study. |
| Family history of PD shows a very high OR (~22). Please check for possible over-adjustment or collinearity in the model. | We thank the reviewer for this comment. The odds ratio of approximately 22 for family history of primary dysmenorrhea represents an unadjusted estimate derived from univariate analysis. Family history did not meet the predefined inclusion criteria for the multivariable model and was therefore not retained in the adjusted analysis. Consequently, this estimate is not subject to over-adjustment or multicollinearity within the multivariable model.  Assessment of multicollinearity was performed only among covariates that advanced from univariate screening to the multivariable model, consistent with standard modeling procedures. Since family history was not included in the multivariable model, collinearity diagnostics were not applicable to this variable. The large unadjusted effect size likely reflects a strong crude familial association with primary dysmenorrhea, as reported in prior literature. We have clarified these points in the manuscript to improve transparency. |
| The comparison with prevalence in Iran (85%), India (75%), etc., is useful, but note differences in measurement tools and age ranges where possible. | We thank the reviewer for this suggestion. We have revised the manuscript to acknowledge that differences in measurement tools, age ranges, and study populations may partly account for the observed variations in prevalence across countries. This clarification has been added to the relevant section to ensure cautious interpretation of cross-country comparisons. |
| The public health implications are well laid out, but consider adding a sentence on how interventions could be delivered given the high out-of-school rate (e.g., community health workers, mobile clinics). | We thank the reviewer for this insightful suggestion. We have revised the Discussion to include a sentence highlighting that, given the high proportion of out-of-school girls, interventions could be delivered through community health workers, mobile clinics, or other community-based platforms to ensure broad reach and accessibility. |
| Minor grammatical edits needed (e.g., “post-menarchael” → “post-menarcheal”; “infrequent pad changing (>6 hours)” could be phrased more clearly as “changing pads less frequently than every 6 hours”). | We thank the reviewer for pointing out these minor grammatical issues. The manuscript has been revised accordingly: “post-menarchael” has been corrected to “post-menarcheal,” and “infrequent pad changing (>6 hours)” has been rephrased as “changing pads less frequently than every 6 hours” for clarity. |
| Ensure consistency in reporting confidence intervals: sometimes written as [54–66], elsewhere as [54% to 66%]. | We thank the reviewer for highlighting this issue. We have carefully reviewed the manuscript and standardized the reporting of confidence intervals throughout. All CIs are now presented consistently in the format “[lower bound%–upper bound%]” to improve clarity and uniformity. |
