# Supplementary material for: Prevalence of primary dysmenorrhea and its associated factors among adolescent girls in a rural setting of Pakistan
Source: PLOS Glob Public Health. 2026 Mar 26;6(3):e0006162. doi: 10.1371/journal.pgph.0006162 (PMC13020788; doi:10.1371/journal.pgph.0006162)
Supplement: Attachment — Submitted filename: R2_response sheet.docx [file pgph.0006162.s005.docx]

Dear Editor,

We sincerely apologize for the oversight in our previous revision. Although corrections to the references had been made, the updated bibliography was not uploaded with the revised manuscript. We have now carefully reviewed and corrected all in-text citations and their associated references, including the issue noted with reference 11. In addition, we have ensured that all statements are supported by relevant papers, and we have addressed Reviewer 1’s concern regarding the citation related to Omega-3 and primary dysmenorrhea.

Please find attached the updated response document and revised manuscript

Sincerely,
Dr Jai K Das

Associate Director

Institute for Global Health and Development

Associate Professor

Department of Paediatrics and Child Health

Aga Khan University, Karachi, Pakistan

Email: [jai.das@aku.edu](mailto:jai.das@aku.edu)

Response Sheet

| Reviewer 1’s comments |  |
| --- | --- |
| Comment | Response |
| You have made substantial and thorough revisions in response to likely reviewer comments. The manuscript is now clearer, more methodologically transparent, and better contextualized. Key issues have been addressed comprehensively.  One of my suggestions have not been considered: I suggested that to use the references for each nutritional items that you have listed. In Omega-3 fatty acid cite this article: Effect of Clupeonella grimmi (anchovy/kilka) fish oil on dysmenorrhea https://doi.org/10.26719/2010.16.4.408. It is a valuable reference and relevant to the introduction section. Please add the same reference. | We apologize for the oversight in the previous revision. Although we had made the correction, the updated bibliography was not uploaded with the revised manuscript. This has now been rectified: the bibliography has been updated to include the corrected citation, ensuring consistency between the in-text references and the reference list. We have also highlighted the updated references. |
