# Supplementary material for: Prevalence of primary dysmenorrhea and its associated factors among adolescent girls in a rural setting of Pakistan
Source: PLOS Glob Public Health. 2026 Mar 26;6(3):e0006162. doi: 10.1371/journal.pgph.0006162 (PMC13020788; doi:10.1371/journal.pgph.0006162)
Supplement: Attachment — Submitted filename: V1_R2 response sheet.docx [file pgph.0006162.s006.docx]

Dear Editor,

We are pleased to resubmit our revised manuscript entitled **“*Prevalence of Primary Dysmenorrhea and Its Associated Factors among Adolescent Girls in Rural Sindh, Pakistan”*** for further consideration in PLOS Global Public Health.

We sincerely thank you and the reviewers for the thoughtful and constructive feedback provided during the review process. We have carefully addressed all comments, including those from Reviewer 3, and have revised the manuscript accordingly. In particular, we have strengthened the conceptual framing of the study, clarified methodological procedures (including outcome operationalization, measurement of lifestyle factors, and multiple imputation procedures), refined statistical justifications, and revised interpretive language to avoid causal overstatement. We have also incorporated additional clarifications in the Discussion to ensure balanced and cautious interpretation of findings.

This study investigates the burden of primary dysmenorrhea among adolescent girls in rural Sindh and examines sociodemographic, nutritional, and lifestyle factors associated with this condition. Despite its substantial implications for adolescent well-being, school participation, and quality of life, dysmenorrhea remains under-researched in low-resource rural contexts. Our findings contribute context-specific evidence that can inform culturally appropriate interventions, strengthen adolescent reproductive health programming, and guide policy development in Pakistan and similar settings.

We believe this manuscript remains well aligned with the scope and mission of PLOS Global Public Health because:

- It addresses an underrepresented dimension of adolescent health in rural South Asia, contributing to equity in global health research.
- It provides actionable evidence relevant to community-based health promotion and menstrual health policy initiatives.
- It amplifies data from a rural population that is often underrepresented in the scientific literature.

We have also carefully reviewed and corrected all references and in-text citations to ensure accuracy and completeness.

The manuscript is original, has not been published elsewhere, and is not under consideration by another journal. All authors have reviewed and approved the revised version and consent to its submission.

We appreciate your time and consideration and look forward to your response.

Sincerely,
Dr Jai K Das

Associate Director

Institute for Global Health and Development

Associate Professor

Department of Paediatrics and Child Health

Aga Khan University, Karachi, Pakistan

Email: [jai.das@aku.edu](mailto:jai.das@aku.edu)

Response Sheet

| Reviewer 3’s comments |  |
| --- | --- |
| Comment | Response |
| Lines 57–62  The manuscript would benefit from a clearer articulation of the specific characteristics of rural Pakistan that justify a separate investigation. Evidence from urban settings in Pakistan may not be generalizable to rural contexts due to substantial differences in socioeconomic conditions, educational access, healthcare availability, cultural norms, and dietary patterns. Strengthening this paragraph by explicitly explaining why findings from urban populations cannot be extrapolated to rural adolescents would improve the study rationale and contextual relevance. | We thank the reviewer for this insightful comment. We have revised Lines 57–62 to more clearly articulate why findings from urban and school-based populations in Pakistan may not be generalizable to rural adolescents. Specifically, we now highlight key contextual differences related to socioeconomic conditions, educational access, healthcare availability, cultural norms surrounding menstruation, and lifestyle factors that justify a separate investigation of rural populations. This revision strengthens the study rationale and improves contextual relevance. |
| Lines 63–72  The discussion on BMI and dysmenorrhea should be strengthened by explicitly acknowledging the existing inconsistency in the literature. Previous studies report conflicting associations between BMI and dysmenorrhea (protective, harmful, or null), and this heterogeneity warrants clearer discussion. Highlighting these inconsistencies would better justify the need for further investigation and contextualize the current findings within the broader evidence base. | We thank the reviewer for highlighting the heterogeneity in the literature on BMI and primary dysmenorrhea. We would like to clarify that this inconsistency is explicitly acknowledged and discussed in the Discussion section, where we note that our findings demonstrate a protective association of higher BMI, contrast these results with studies reporting increased menstrual pain at higher BMI levels, and emphasize the conflicting nature of existing evidence. We further discuss potential region-specific explanations, including the use of South Asian BMI cut-offs and dietary factors, and explicitly state that these mechanisms are hypothetical and warrant further investigation. Given that Lines 63–72 are intended as background context, we believe that an in-depth discussion of heterogeneity is appropriately placed in the Discussion and is already adequately addressed there (line 312-325). |
| Lines 49–56  This paragraph currently provides descriptive definitions of primary and secondary dysmenorrhea but contributes limited conceptual value. If retained, it should be strengthened by explicitly discussing the physical, psychological, educational, and social consequences of both PD and SD among adolescent girls. Emphasizing impacts such as school absenteeism, reduced quality of life, and psychosocial stress would better establish the urgency and public health relevance of studying dysmenorrhea. | We thank the reviewer for this valuable suggestion. We have revised Lines 49–56 to strengthen the conceptual contribution of this paragraph by explicitly highlighting the physical, psychological, educational, and social consequences of both primary and secondary dysmenorrhea among adolescent girls. The revised text now emphasizes impacts such as impaired daily functioning, school absenteeism, reduced academic performance, diminished quality of life, and psychosocial stress, thereby reinforcing the public health relevance and urgency of studying dysmenorrhea in this population. |
| Lines 66–68  The manuscript introduces omega-3 fatty acids, magnesium, vitamin B6, calcium, and iron as relevant nutritional factors, but the empirical or mechanistic basis for their inclusion is not sufficiently explained. The authors should briefly summarize existing evidence or biological mechanisms linking these micronutrients to dysmenorrhea (e.g., inflammation, prostaglandin synthesis, neuromuscular function). In addition, it would strengthen the argument to explain why these nutrients are likely to be deficient in rural settings, including discussion of limited dietary diversity, reliance on staple-based diets, low animal-source food consumption, and constrained food access. | We thank the reviewer for this constructive suggestion. We have revised Lines 66–68 to briefly clarify the biological relevance of omega-3 fatty acids, magnesium, vitamin B6, calcium, and iron in relation to dysmenorrhea, highlighting their roles in inflammation, prostaglandin synthesis, and neuromuscular function. We also explicitly explain why deficiencies in these micronutrients are more likely in rural settings, including limited dietary diversity and constrained access to nutrient-rich foods. Detailed discussion of empirical evidence and mechanistic pathways is provided in the Discussion section, where these associations are examined in depth in relation to our findings. |
| Lines 68–70  The statement suggesting reduced physical activity influenced by urbanization requires clarification. Many studies report higher levels of physical activity in rural populations due to domestic and agricultural labor. The authors should clarify whether urbanization in rural Pakistan refers to lifestyle transitions (e.g., increased screen time, reduced recreational activity, schooling patterns) and support this claim with references or contextual explanation. | We thank the reviewer for this important clarification. We have revised Lines 68–70 to avoid implying that rural populations uniformly experience lower physical activity due to urbanization. Instead, we now describe context-specific lifestyle transitions in rural areas, including increased screen time, changing schooling patterns, and shifts in daily routines that may influence physical activity and sleep behaviours. This revision provides a more accurate and nuanced contextual explanation. |
| Line 145 The manuscript states that primary dysmenorrhea was defined as menstrual pain “without underlying reproductive disease,” but it is unclear how underlying gynecological conditions were excluded. Please clarify whether this was based solely on self-report, symptom screening, medical history, or any clinical assessment, and discuss the implications of potential misclassification. | W e thank the reviewer for this important methodological clarification. Primary dysmenorrhea was operationalized based on self-reported menstrual pain. No clinical examination or diagnostic screening was conducted to exclude underlying gynecological conditions, and classification relied on participant self-report. We have revised the Methods section to clarify this operational definition. We have also expanded the Limitations section to acknowledge the possibility of misclassification between primary and secondary dysmenorrhea and its potential impact on prevalence estimates and associations. |
| Line 168 Please clarify how sleep duration was assessed. Was sleep measured using a validated questionnaire, structured interview, or a single self-reported question? If self-report was used, this limitation should be acknowledged. | We thank the reviewer for this important clarification. We have revised Lines 169–171 to specify that sleep duration and physical activity were assessed using self-reported items adapted from the HBSC and GSHS instruments with a seven-day recall period. Both of these tools are already cited in the manuscript under the section “Data collection tools”. |
| Lines 169–171 It is unclear whether a validated physical activity instrument was used or whether activity levels were assessed through simple recall questions. Please specify the instrument, scoring method, and rationale for the chosen cut-off (≥180 minutes/week). | Physical activity was also assessed using GSHS questionnaire. We have also clarified the scoring method for physical activity, including calculation of weekly minutes and justification for the ≥180 minutes/week cut-off. Although the original item assessed the number of days achieving ≥60 minutes of MVPA, we derived total weekly MVPA by multiplying reported active days by 60 minutes. This transformation did not alter participant responses but expressed them as cumulative weekly minutes to enable categorization according to established weekly physical activity thresholds. This frequency × duration approach is consistent with standard epidemiological practice (IPAQ scoring methods).We have also acknowledged in the Limitations section that both measures were based on self-report and may be subject to recall bias. |
| Lines 198–200 Further clarification is needed on how the multiple imputation process was validated. Please indicate which variables were included in the imputation model, how convergence was assessed, and whether sensitivity analyses were conducted to ensure robustness. | We thank the reviewer for requesting clarification regarding the multiple imputation procedure. Missing data were minimal (2.5%) and limited to nutrient intake variables. Multiple imputation was performed using observed covariates included in the analytical model, and final regression estimates were pooled across imputed datasets using Rubin’s rules, as implemented in Stata’s mi estimate procedure. This approach accounts for both within- and between-imputation variability, ensuring appropriate estimation of standard errors and confidence intervals. Given the very low proportion of missing data, extensive additional sensitivity analyses were not expected to materially alter results. The Methods section has been revised to clarify these details. |
| Line 206  The use of a significance threshold of p < 0.1 in multivariable analysis is uncommon in population-based epidemiological studies. A clear methodological justification is required, including whether this threshold was chosen due to exploratory objectives, sample size considerations, or concerns about statistical power. | We thank the reviewer for raising this important methodological point. While p < 0.05 is conventionally used in epidemiological studies, this analysis was exploratory and conducted in a previously understudied rural population. In addition, primary dysmenorrhea was assessed using a single self-reported question, which may introduce measurement variability. To reduce the risk of Type II error and avoid overlooking potentially meaningful associations under these conditions, a significance threshold of p < 0.10 was adopted in the multivariable analysis. Effect sizes and 95% confidence intervals are reported to emphasize magnitude and precision rather than sole reliance on p-values. This rationale has now been clarified in the Methods section. In addition, certain references have supported the idea of keeping p<0.1 to prevent type II error:   1. Miller J, Ulrich R. The quest for an optimal alpha. PloS one. 2019 Jan 2;14(1):e0208631.   In addition, there are certain studies who utilized a level of significance of p<0.1:   1. Akseer N, Rizvi A, Bhatti Z, Das JK, Everett K, Arur A, Chopra M, Bhutta ZA. Association of exposure to civil conflict with maternal resilience and maternal and child health and health system performance in Afghanistan. JAMA Network Open. 2019 Nov 8;2(11):e1914819. 2. Salles GF, Bloch KV, Cardoso CR. Mortality and predictors of mortality in a cohort of Brazilian type 2 diabetic patients. Diabetes Care. 2004 Jun 1;27(6):1299-305. |
| Lines 264–267  The authors report that each 10 g increase in carbohydrate intake slightly increased the odds of PD. It would be informative to clarify whether this association differs by BMI category (underweight, normal weight, overweight). Additional stratified analysis or discussion would help interpret whether this finding applies across all nutritional statuses or specific subgroups. | We thank the reviewer for this thoughtful suggestion. In our analysis, BMI and carbohydrate intake were included as independent covariates in the multivariable model, and the reported odds ratio for carbohydrate intake reflects the overall association across the study population. Assessment of interaction or stratified analyses by BMI category was not part of the pre-specified analytical plan and may be limited by sample size within strata. We agree that potential effect modification by nutritional status is an interesting hypothesis and have now acknowledged this as a direction for future research in the Discussion. |
| Lines 296–297 Caution is warranted when describing overweight/obesity and cloth pad reuse as “protective” factors. These statements should be immediately accompanied by nuanced discussion and clear acknowledgment of alternative explanations, residual confounding, and potential misinterpretation, particularly given the public health implications. Integrating the interpretation directly with the results presentation would improve clarity and reduce the risk of overstatement. | We thank the reviewer for this important point. We agree that causal terminology should be avoided when interpreting observational findings. We have revised the text to replace “protective factors” with “associated with lower odds” and added clarifying language to emphasize cautious interpretation. This change ensures that results are presented as associations rather than causal effects. |
| Lines 298–305  The explanation of birth order effects relies heavily on speculative cultural interpretations. While the proposed psychosocial mechanisms are interesting, they are not sufficiently supported by empirical data. The authors should either provide supporting references or clearly tone down speculative language and present these explanations as hypotheses requiring further investigation. | We thank the reviewer for this helpful observation. We agree that the proposed mechanisms should be clearly framed as hypotheses rather than established explanations. We have revised the text to soften the language and explicitly present these interpretations as speculative, while retaining them as potential avenues for future research. |
